# Supplementary material for: CTRP9 Mediates Protective Effects in Cardiomyocytes via AMPK- and Adiponectin Receptor-Mediated Induction of Anti-Oxidant Response
Source: Cells. 2020 May 15;9(5):1229. doi: 10.3390/cells9051229 (PMC7291146; doi:10.3390/cells9051229)
Supplement: Supplementary file 1 [file cells-09-01229-s001.pdf]

Supplement to: Niemann et al.: CTRP9 mediates protective effects in cardiomyocytes via AMPK- and adiponectin receptor-mediated induction of anti-oxidant response

Tables

Supplementary Table 1: siRNA

	sense strand sequence
**AdipoR1 rat**	5′-GGCUCUAUUACUCCUUCUATT-3′
**AdipoR2 rat**	5′-AAAUUGGAUUACUCUGGAUTT-3′
**T-cadherin rat**	5′-CGGGCUGGCUGAGUAUUAATT-3′
**calreticulin rat**	5′-CCGGUGUAAGGAUGAUGAATT-3′
**gC1qbp rat**	5′-CGGCACGGAGGCUAAAUUATT-3′
**alpha 1 AMPK rat**	5′-GGCACCCUCAUAUAAUCAATT-3′
**alpha 2 AMPK rat**	5′-GGCCAUAAAGUGGCAGUUATT-3′
**SIRT3 rat**	5′-GGCCCAAUGUCGCUCACUATT-3

Supplementary Table 2: Primer sequences

	GenBank accession #	Forward Primer	Reverse Primer
**CTRP9 rat**	NM_001191891	GGTGGCTTCTACTGGTTATGGA	CTCTCCACGAATTCCATCCTT
**AdipoR1 rat**	NM_207587	TCCTGAGCGCTTCTTTCCTG	AGAAGGGAGTCGTCGGTACA
**AdipoR2 rat**	NM_001037979	GACGGGCAACATTTGGACAC	AAAGGCAGAGAATGGCTCCC
**T-Cadherin rat**	AF494095	TGAACAGCGATGGCACCTTA	CCCCGACAATCACGAGTTCT
**Trx1 rat**	NM_053800	GCTGATCGAGAGCAAGGAAG	TGGCAGTCATCCACGTCTAC
**Trx2 rat**	NM_053331	GAGACACCAGTTGTCGTGGA	TGGCAAGGTCTGTGTGATCG
**SOD1 rat**	NM_017050	GTGGCCAATGTGTCCATTGAA	AATCACACCACAAGCCAAGC
**SOD2 rat**	NM_017051	ATCTGAACGTCACCGAGGAG	GGCTCAGGTTTGTCCAGAAAAT
**GPX1 rat**	NM_030826	TCGGACATCAGGAGAATGGCAAGA	GATCGTCACTGGGTGCTGGC
**GPX2 rat**	NM_183403	TGGCTTACATCGCCAAGTCT	CGAACTGGTTGCAAGGGAAG
**catalase rat**	NM_012520	CCTGACATGGTCTGGGACTT	CAAGTTTTTGATGCCCTGGT
**Nox2 rat**	NM_023965.1	CAGTGTGTCGGAATCTCCTCT	ACCACTCCACGTTGAACAGA
**Nox4 rat**	NM_053524.1	TAACCTCAACTGCAGCCTTATC	CTTTTATCCAACAATCTCCTGGTTCTC
**p22 phox rat**	NM_024160.1	ATCAAGCAGCCACCTACCAA	ATTGGGTTAACCTGGGGTCC
**p47 phox rat**	NM_053734.2	CCTGTCGGAGAAGGTGGTCTA	GGAGGTGAGGGATGACTCTGT
**18S rRNA**	NR_046237	GTTGGTGGAGCGATTTGTCTG	GCTCAATCTCGGTGGCTGA

Figures

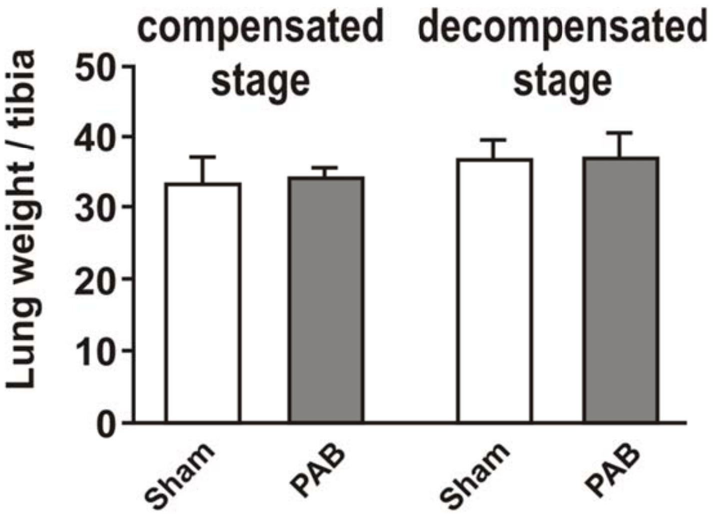

Supplementary Figure 1: Lung weight. Changes in lung weight (weight in mg / tibia in mm) in sham animals and PAB animals 7 weeks after surgery (compensated stage) or 22 weeks after surgery (decompensated stage). Data are mean±SEM, n=14 animals per group.

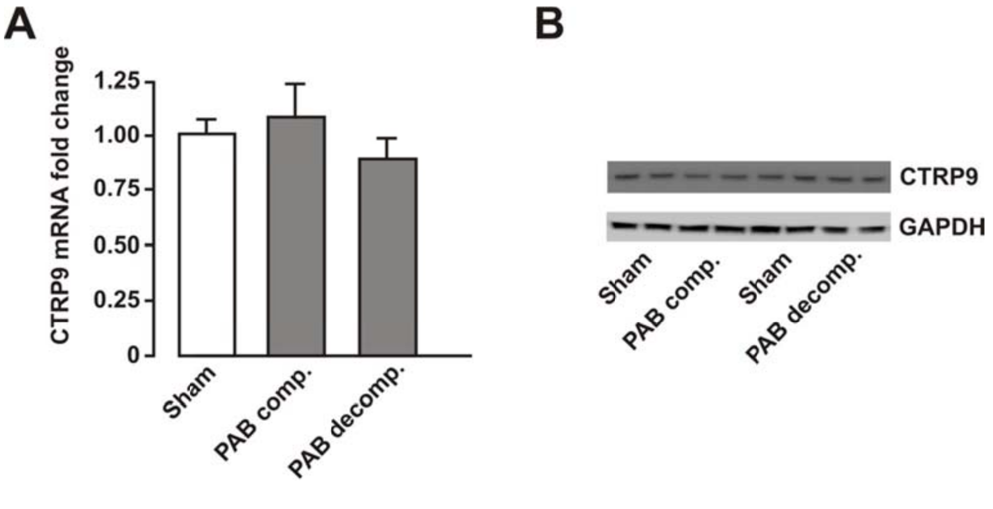

Supplementary Figure 2: CTRP9 expression in RV endothelial cells. A: Changes in CTRP9 mRNA expression in ECs from sham animals and PAB animals at the stage of compensated hypertrophy (PAB comp.) or RV decompensation (PAB decomp.) as determined by real-time PCR. **B:** Changes in CTRP9 protein expression in ECs from sham animals and PAB animals as determined by Western blot. GAPDH served as a loading control. n=6 animals per group. Data are mean±SEM, n=5 per group.

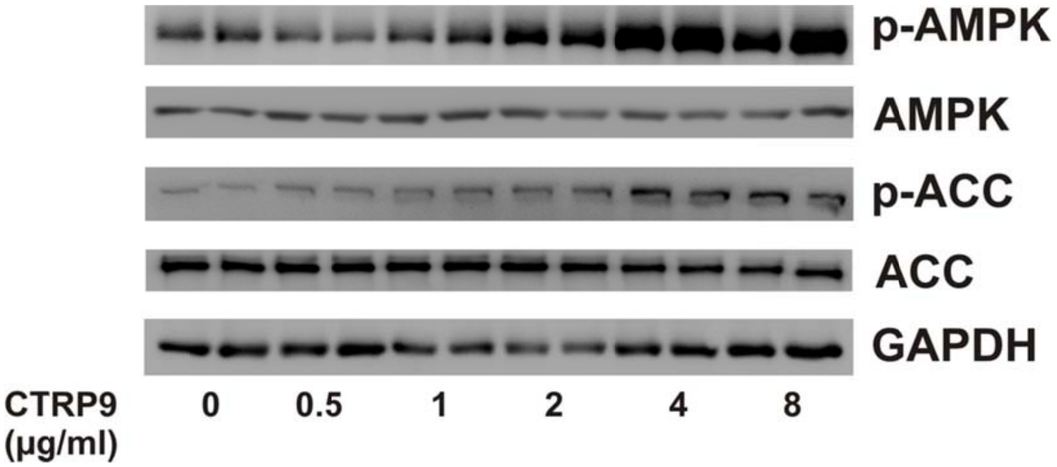

Supplementary Figure 3: Concentration-dependent activation of AMPK and ACC in cardiomyocytes. Adult rat cardiomyocytes were treated with the indicated amount of CTRP9 for 15 min and activation of AMPK and ACC was determined by Western Blotting. GAPDH, total AMPK and total ACC served as a loading control. n=6 samples per group, 3 independent experiments.

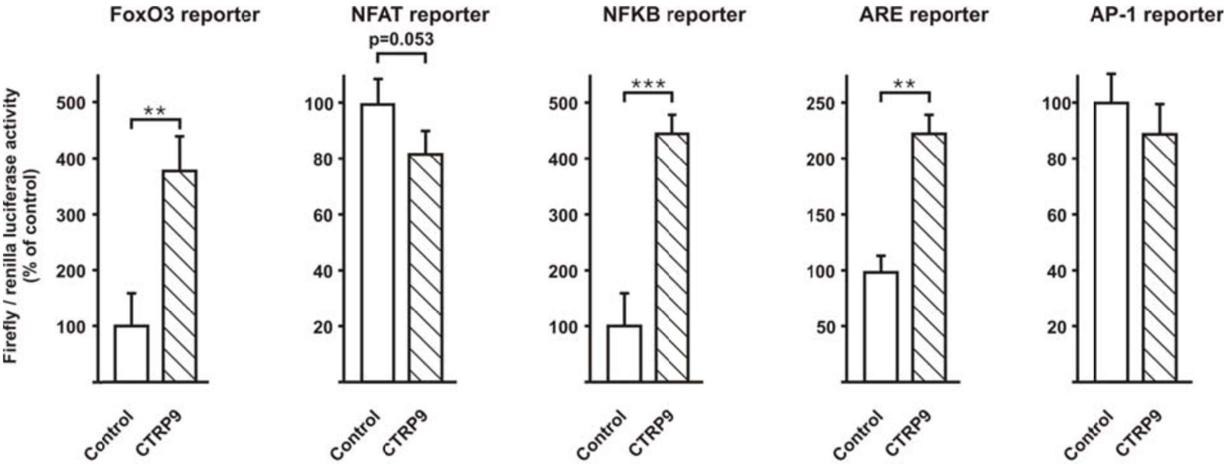

Supplementary Figure 4: Transcription factor activation in response to CTRP9. H9C2 cardiomyoblasts were transfected with a renilla luciferase reporter gene (control vector for normalization) and firefly-luciferase reporter genes containing binding sites of various transcription factors including Foxo3a, NFAT, NFkB, Nrf2/ARE or AP-1. Luciferase activity was measured 48 hours after transfection and 24 hours of stimulation with CTRP9 (4µg/ml). Data from 4 independent experiments. Data are mean±SEM, **: $p<0.01$, ***: $p<0.001$, n=12 per group.
